# Agrobiotechnology Goes Wild: Ancient Local Varieties as Sources of Bioactives

**DOI:** 10.3390/ijms19082248

**Published:** 2018-08-01

**Authors:** Roberto Berni, Claudio Cantini, Marco Romi, Jean-Francois Hausman, Gea Guerriero, Giampiero Cai

**Affiliations:** 1 Department of Life Sciences, University of Siena, via P.A. Mattioli 4, 53100 Siena, Italy; berni10@student.unisi.it (R.B.); marco.romi@unisi.it (M.R.); 2 Trees and Timber Institute-National Research Council of Italy (CNR-IVALSA), via Aurelia 49, 58022 Follonica (GR), Italy; cantini@ivalsa.cnr.it; 3 Research and Innovation Department, Luxembourg Institute of Science and Technology, 5 Avenue des Hauts-Fourneaux, L-4362 Esch/Alzette, Luxembourg; jean-francois.hausman@list.lu

**Keywords:** agrobiodiversity, ancient varieties, bioactives, nutraceuticals, -omics

## Abstract

The identification and use of species that have best adapted to their growth territory is of paramount importance to preserve biodiversity while promoting sustainable agricultural practices. Parameters including resistance to natural conditions (biotic and abiotic risk factors), biomass and fruit productivity, and phytochemical content with nutraceutical potential, could be used as quantitative markers of the adaptability of plants to wild environments characterized by minimal human impact. Ancient varieties, which are plant varieties growing in regional territories and not destined for market distribution, are a source of unique genetic characters derived from many years of adaptation to the original territory. These plants are often more resistant to biotic and abiotic stresses. In addition, these varieties have a high phytochemical (also known as bioactives) content considered health-beneficial. Notably, the content of these compounds is often lower in commercial cultivars. The use of selected territorial varieties according to the cultivation area represents an opportunity in the agricultural sector in terms of biodiversity preservation, environmental sustainability, and valorization of the final products. Our survey highlights the nutraceutical potential of ancient local varieties and stresses the importance of holistic studies (-omics) to investigate their physiology and secondary metabolism.

## 1. Introduction

The 1992 Rio de Janeiro environmental conference defined biodiversity as the “variability of organisms living in terrestrial, marine, and aquatic ecosystems and the biological complexes of which they are a part”. Variability is defined considering three fundamental parameters: ecosystems, species, and genetic traits. These three aspects are directly related and responsible for the expression of multiple morphological characteristics in species belonging to different habitats [1]. A large biological diversity means a wider genetic reservoir that helps living organisms respond to the environmental constraints that the various ecosystems face and to which the most suited organisms have adapted. Environmental changes and anthropogenic activities have deeply impacted ecosystems, with repercussions in agriculture and consequent loss in genetic diversity in varieties [2]. More specifically, in agriculture, human activity is certainly the main cause of environmental alterations: industrial pollution, deforestation, and the introduction of genetically modified organisms has led to imbalances in ecosystems, translating into a loss in genetic agrobiodiversity. Indeed, agricultural practices have been directed for years toward mass production that have caused the extinction of species that are not particularly profitable in terms of productivity [3].

Nowadays, biodiversity is recognized as a fundamental resource for biological systems; nations around the world have committed to safeguarding biodiversity through laws and regulations. In this respect, Italy has defined specific rules for each national region [4]. More specifically, Fideghelli et al. [5] defined the Italian regional legislative model as an example of biodiversity protection and the safeguard of agricultural species of interest in the European context. As far as agrobiodiversity is concerned, each Italian region has a database that contains the phenotypic and genetic characteristics [6] of all the indigenous varieties in the territory. Due to the application of local laws, recovery activities commenced that spread from provincial campaigns to regions and, eventually, to the entire country. The collected data reported the immense heritage of ornamental and fruit plants cultivated in the past and memories of their uses in the community. The uses were multiple: for the consumption of fruits, for wood and fodder, to signal borders, provide shelter to birds, and to support other plants and wild animals [7]. These plants represent a cultural heritage linked to local customs and traditions that directly involved society. Furthermore, these varieties have unique genetic characteristics resulting from many years of adaptation to the native territory.

The potential for variability in the plant germplasm has been well studied and it is thought that, over the centuries, the varieties have accumulated mutations in the genome that could provide resistance to various environmental conditions. Lenne et al. [8] reported the use of wild germplasm as a potential resource to improve the disease resistance of cultivars, with a focus on food crops. In particular, this work reported that the wild germplasm is more resistant to environmental factors and identified its broad spectrum of genetic resistance mechanisms as a source of characters for the improvement of cultivars.

Several reviews have been published on crop wild relatives (CWRs), which are the wild relatives of domesticated crops [9], and landraces (LRs), which are traditional varieties maintained by traditional farming systems [10], and their biotechnological potential for crop improvement [11,12,13]. The identification of CWR and LR genes that confer increased resistance to exogenous constraints is an effective strategy preceding biotechnological and breeding programs for the improvement of several crop species. For example, a study identified a resistance gene—a nucleotide-binding site leucine-rich repeat (NBS-LRR)—against the root-knot nematode *Meloidogyne* spp. from the wild relative of eggplant, *Solanum aculeatissimum* [14], and discussed the biotechnological potential of transferring the gene to cultivated eggplants to create more resistant introgression lines. Several other examples exist in the literature. A previous study identified the *Rz2* gene, which is a nucleotide binding coiled-coil leucine-rich repeat, conferring rhizomania resistance in a wild population of beets (*Beta vulgaris* ssp. *maritima*). The study used mapping-by-sequencing to identify the *Rz2* gene and further validated its role by performing RNA interference [15].

Among the different plant families representing important staple food crops, *Poaceae* include both wild relatives and landraces showing enhanced stress tolerance traits with respect to domesticated counterparts [16]. In this respect, a study showed how the ectopic expression of a *PYL3* gene encoding an ABA receptor, from the drought-tolerant rice landrace Nagina 22, enhanced cold and drought resistance in thale cress [17].

Notably, stress resistance is controlled by polygenic traits, which are difficult to introgress in domesticated crops. An alternative approach is de novo domestication, where the editing of domestication genes is performed in CWRs. Researchers have analyzed how to obtain the ideotype (i.e., the archetype of the cultivated plant both in terms of vegetative and reproductive growth) of tomato by applying gene editing on a CWR (*Solanum galapagense*) [18]. Both the studies addressing the potential of resistance gene mining and those reporting gene editing strategies highlight the enormous importance of studying and preserving the germplasm of CWRs.

Other scientific reports have correlated the availability of different genetic resources and adverse environmental conditions by focusing on climatic events that could negatively affect the agronomical harvests (abiotic stress). These studies have focused on abiotic factors, such as waterlogging, drought, and temperature fluctuations related to climate change [19,20]. Studies in the literature have reported that, in many cases, the most efficient and least costly response to stress is the use of new adapted varieties [21] and, in this respect, a large availability of germplasm could facilitate the task.

Ancient native varieties, as discussed above for CWRs and LRs, meet these requirements: the years of adaptation to the territory have selected these plants. Safeguarding these varieties as possible sources of characters involved in the resistance to environmental stress is therefore of fundamental importance. Some studies have quantified bioactives in ancient species of both herbaceous and woody horticultural crops (Table 1). For example, spelt varieties, which are alternatives to wheat, were shown to possess all high total antioxidant activity. More specifically, the high amount of bioaccessible and bioavailable free phenolic acids, together with the added environmental benefit of growing spelt over wheat (no need for pesticides, lower nitrogen fertilization) make the use of spelt-derived products highly attractive for consumers [22]. However, some studies have found few differences between ancient and modern species [23] in terms of bioactives. The exception is for carotenoids, and lutein in particular, which was reported to be much higher in einkorn with respect to bread wheat [23]. The higher content of carotenoids in einkorn was confirmed in another study that compared ancient wheat populations from Italy, Turkey, Georgia, Bulgaria, and Armenia [24]. In the same publication, considerable within-species variability was found, suggesting the potential interest in studying the chemical profile of specific compounds in individual populations.

Concerning woody species, a study on the fatty acid content of ancient olive trees from Southern Italy was completed [27]. The 10 most commonly cultivated varieties, together with 27 ancient and recently-introduced varieties, were analyzed using genotyping and fatty acid composition. The results reported high amounts of polyunsaturated fatty acids (PUFAs) in almost all the old accessions Salella and highlighted the interest in studying and introducing such varieties into olive collections.

Ancient local varieties of apple from Croatia were shown to possess high polyphenol content (flavanols and phenolic acids) in the flesh, whereas no major differences were observed in the peels [29]. The study highlighted non-commercial apple varieties as potential sources of polyphenols.

Ancient grape cultivars from Eastern Turkey also showed high phenolic compound levels, with different rankings among the cultivars studied, depending on the class of phenolics [28].

A large body of evidence in the literature highlights the potential bioactive content in ancient varieties of herbaceous and woody species.

In the present review, we emphasize the agrobiotechnological value of ancient local varieties as under-utilized alternatives to commercial varieties. The nutraceutical aspect of autochthonous plants, which is the manifestation of specific genetic traits, is discussed, given their potential use as components of functional foods.

## 2. Harnessing the Power of Plant Secondary Metabolites: Functional Foods and Human Health

Plants produce a rich palette of phytochemicals (plant secondary metabolites) that have bioactivity and are hence of industrial interest [30]. Ancient plant varieties are a source of bioactive compounds and can therefore contribute to the development of nutraceuticals. The interest of consumers towards phytochemicals with beneficial health effects has led nutritionists to research and develop new foods with high nutraceutical content. The word “nutraceutical” was coined by the nutritionist and biochemist Stephen DeFelice in 1989 by merging the terms “nutrition” and “pharmaceutical”, thus defining a product with the properties of food, but with added beneficial health properties. More specifically, according to Pandey et al. [31], “nutraceutical is any substance present in a food, or part of a food, that has beneficial health effects, including the prevention or treatment of diseases”. Nutraceutics investigates components or active ingredients with the aim of developing new food products capable of improving the psycho-physical state and reducing the susceptibility to diseases through prevention [32]. The term “functional food” was first defined in Japan in the 1980s to indicate a food product enriched with natural ingredients, generally absent or present at reduced concentrations in other foods, which has the ability to positively affect human physiology. According to the International Life Science Institute, functional foods should be consumed by healthy subjects as an integral part of a proper diet [33].

These foods are interesting for their nutraceutical characteristics because of their phytochemicals, which are secondary metabolites such as polyphenols, terpenoids, and alkaloids. Plant bioactives are gaining ever-increasing interest given their beneficial impact on human health. For example, the consumption of microgreens (i.e., plants originating from the seeds of vegetables and grains), which are rich in carotenoids, is being considered during space missions as a component of the Life Support System to protect against the damages induced by the extreme environment encountered during space missions, including radiation, oxidative stress, and physical and mental stress [34].

Polyphenols are plant secondary metabolites that play a fundamental role in a wide range of functions: they are responsible for the color of flowers, fruits, and seeds; they act as signaling molecules in plant-microorganism interactions; they provide protection from ultraviolet light, and defend the plant from pathogens and predators [35]. The multifunctionality of polyphenols is due to their distribution in different tissues and organs of plants and at different concentrations. From a biochemical point of view, polyphenols (e.g., phenolic acids, flavonoids, lignins, and stilbenes) are a class of organic compounds including several aromatic rings associated with different phenolic groups and their properties include a strong antioxidant activity [36].

Terpenes, such as hemiterpenes, monoterpenes, sesquiterpenes, diterpenes, sesterterpenes, triterpenes, sesquarterpenes, tetraterpenes, polyterpenes, and norisoprenoids, are secondary metabolites composed of more isoprenic units. Plants produce terpenes through the reaction of several units of isopentenyl pyrophosphate (IPP) and dimethylallyl pyrophosphate (DMAPP) [37]. Terpenes have antioxidant activity related to their chemical structure [38]. For example, the radical scavenging properties of inuroyleanol are due to the presence of two hydroxyl groups and one methoxyl on the aromatic ring [38]. Terpenes also have anti-inflammatory and anti-tumor activity, and exert positive effects on neuronal health. Since they are the key components found in forest aerosols, many countries have adopted “forest bathing” as a therapeutic means to improve physical and mental well-being [39].

Vitamins are indispensable micronutrients for our bodies. These molecules also have antioxidant activity. In vitro studies demonstrated that vitamin consumption decreases the risks of chronic disease by acting as direct antioxidants, like vitamin E [40], and as electron donors, as is the case for vitamin C [41]. Compounds with anti-oxidant and anti-inflammatory activity have received considerable attention as important molecules in the diet. An antioxidant is any substance which, although present in concentrations much lower than a given oxidizable substrate, significantly delays or prevents the oxidation of that substrate [42]. The antioxidant capacity of polyphenols depends on the number and position of hydroxyl groups bound to the aromatic ring and, in general, on the geometry of the molecule that allows them to neutralize free radicals and chelate metals [43]. In human cells, polyphenols scavenge oxygen free radicals, such as reactive oxygen species (ROS).

Increasing ROS content in the cell can cause damage to biomolecules, such as lipids, proteins, and DNA, as well as alter membrane functions [44]. Oxidative stress and/or non-detoxification of ROS seem to be the main causes of diabetes, cardiovascular diseases, cancer, Alzheimer’s, and other neurodegenerative diseases [45]. High levels of phenolic compounds, terpenes, alkaloids, and vitamins are commonly found in onions, carrots, potatoes, and tomatoes, which are different foods that are included in the daily diet [46]. Due to the presence of these molecules, these foods can be classified as functional foods, meaning they can maintain the state of physical well-being. Today, new natural products that contain high levels of bioactive compounds and that can be consumed through food are being researched and studied. Studies on the effects of drugs in therapy for patients with cardiovascular disease showed that nutraceuticals increase the reactivity of the drugs and the efficiency of the treatment [47]. Consequently, the presence of this type of food in the human diet is important. The priority is therefore to search for foods with a high content of molecules that can be studied in the medical and pharmaceutical fields [48], to potentially make these products more accessible to consumers.

Since compounds that have nutraceutical potential are, in many cases, involved in plant defense mechanisms against (a)biotic stress, their abundance is directly related to the ability of a plant to react to the environment. Ancient varieties have developed stress response mechanisms enabling them to thrive in a wild context. These stress response mechanisms are more efficient than those of cultivated varieties and may be correlated with an increased content of bioactive molecule. Several authors have studied the content of antioxidants and polyphenols in ancient varieties and reported higher values than the commercial cultivars of the same species. In particular, Iacopini et al. [49] suggested ancient apples as a potential future natural resource for food science and nutrition. Ancient varieties can promote innovation in the science of nutrition; they can be used in the development of commercial lines of fruits with high bioactive power, thus promoting a wider range of local products that can be offered to the consumer. Studies have proposed “green technologies”, which are agricultural approaches intended to limit the effects of human activities on the environment, as a possible method to diversify the current crop and food product markets [50]. As far as sustainable development is concerned, these applications are able to direct agriculture toward processes that guarantee the progress of economic production under safe and high-quality standards.

A minimum human processing area is a possible strategy in the sustainable development of agricultural production. Agricultural land with a lower human impact preserves the quality of soils and avoids mineral impoverishment [51]. The problem may be solved with the use of ancient varieties adapted to difficult growth conditions, which would limit the consumption of chemicals and minimize the loss in productivity. Due to these characteristics, ancient local varieties are an important natural resource promoting innovation in the agricultural sector and provide an opportunity to save the territorial biodiversity threatened by intensive agriculture. Sustainability and preservation of local germplasm should be combined for the future sustainable development of agriculture.

## 3. Ancient Native Varieties as a Treasure-Trove of Bioactives: Tuscany

Plant bioactive compounds have been well studied in phytochemistry due to their beneficial health effects and their ability to be introduced in the diet. Scientific interest in the use of compounds from natural sources rather than synthetic compounds has grown [52]. Plant secondary metabolites are involved in defense mechanisms against (a)biotic stresses. Plants evolve these defense mechanisms as adaptations to the habitat of origin that enable survival in a territory subjected to unfavorable conditions [53]. These molecules also confer to the plant the ability to rapidly react to environmental constraints [54]. Humans selected the more productive species, given the commercial needs, and abandoned less productive varieties. These less productive varieties thrive in wild areas, forced to adapt to harsh territorial conditions. The genome of these plants has been shaped by long adaptation to the territory and represents a source of unique characters, such as specific phenotypic traits like shape, color, and biochemical composition of fruits [55]. Most of these components are phenolic molecules, such as hydroxycinnamic acids, flavonoids, and anthocyanins, which are different even among fruits of the same species in many cases [56].

Since the Rio de Janeiro conference, many countries have made efforts to conserve biodiversity. Italy showed significant interest in its own agrobiodiversity through the creation of laws specific for each region. The aim of these laws was to safeguard and enhance the preservation of the ancient plants in the regional territory, preventing the risk of genetic erosion to which some varieties had been subjected. Many recovered ancient crops are of agricultural or forestry interest, depending on the species and variety. The potential use and valorization of these ancient plants has fostered their introduction into the commercial landscape and shifted the purpose of most laws towards the consideration of economic aspects [4].

Tuscany was the first Italian region to introduce a law to preserve biodiversity with the 64/04 law [57]. This law allowed Tuscany to investigate the large regional plant asset. All the recovered species have been classified into a database of phenotypic aspects, which constitute the Tuscan regional germplasm heritage. The Regional Bank of Germplasm protects genetic resources through ex situ conservation (seed banks, collection fields, etc.) and avoids any form of contamination, alteration, or destruction. The Regional Bank is divided into various sections distributed throughout the region, which combine the information into a single database containing the descriptive data of the species, phenotypic and genetic description of plants, and the supporting photos of plants, fruits, and leaves of each variety. The database groups the accessions into five sections: autochthonous animal genetic resources, ornamental and flowering species, forest species, herbaceous and woody species, as well as fruit species (http://germoplasma.regione.toscana.it/index.php?option=com_content&view=article&id=4&Itemid=109).

Some ancient species classified by the Regional Bank sections are now considered an agricultural resource from an ecological point of view. Many Italian regions promote these varieties for commercial purposes. The goal is to manufacture food products deriving from the cultivation of these local varieties. Scientific reports focused on fruits provided interesting values in terms of functional compound content. With respect to functional molecules, the ancient varieties have characteristics that encourage the consumption of their fruits. Other authors have emphasized the unconventional qualitative traits of ancient varieties through sensory analysis; these fruits are different in shape and color from commercial cultivars, display peculiar organoleptic features, and positive nutritional values [58]. Considerable differences were also found in the qualitative and quantitative profiles of the phenolic compounds—a feature sought after by the consumer given the antioxidant potential of these molecules. Commercial fruits conform to the needs of large-scale distribution, but need chemical treatments to achieve maximum production. However, chemicals cause an increase in environmental pollution that threatens agriculture. The use of ancient crops could provide a solution to this problem. Some authors reported data that demonstrate the lower environmental impact of ancient varieties per unit of cultivated land [59].

Extending the study of ancient varieties that are a fundamental component of the territorial biodiversity is essential and their use could represent the next step towards functional foods [29,60]. These plants represent a bridge between the past, in terms of popular culture, and the future, given their possible use in innovative agricultural development and sustainable technologies.

## 4. Importance of -Omics for the Study of Plant Secondary Metabolism in Non-Model Species

Plant bioactives have a signature that changes depending on the species, the growth conditions, and on the organs of the plant [61,62]. This clearly adds further complexity to the study, characterization, and determination of the molecular factors involved in the biosynthesis of secondary metabolites. However, progress in the field of molecular analyses via Next-Generation Sequencing has provided unprecedented analytical depth, thereby offering new perspectives in the study of plant secondary metabolome. -Omic approaches applied to ancient local varieties help explain the mechanism of adaptation to the territory and enable the identification of those varieties expressing the best characteristics for potential commercial exploitation. In this section, we provide examples of holistic approaches (-omics) used in systems biology for the study of secondary metabolism in non-model plants. What can be learned about non-model species can be transferred to native (ancient) plants, which, when compared to traditional commercial plants, could be considered non-model varieties.

The analytical depth of transcriptomics was coupled with elicitation with methyl jasmonate to uncover the genes involved in secondary metabolite biosynthesis in *Lycoris aurea* [63]. Several transcription factors (TFs), WRKY, MYB, and AP2/ERF, and transporters were thus identified. Transcriptomics was also used to unveil the metabolic network underlying secondary metabolite biosynthesis in tea plants [64]. Several TFs were also identified that link flavonoid, caffeine, and theanine biosyntheses. A transcriptomic and proteomic shot-gun approach was used with the ornamental plant *Peperomia obtusifolia* L. to study the production of benzopyrans derived from orsellinic acid, a phenolic acid usually found in fungi [65]. The analyses led to the identification of both the mevalonate and methylerythritol pathways as being active in the leaves, and showed that terpenoid biosynthesis was the pathway with the highest number of enzymes identified.

A merged transcriptomics-metabolomics study in different tissues of Neem identified members of the CYP450 family as responsible for the synthesis of tetranortriterpenoids, thereby paving the way to future functional studies [66].

Insightful are the studies performed on isolated plant trichomes. Since glandular trichomes are the cell “factories” where secondary metabolites are produced and accumulated, by isolating them and analyzing their transcriptome, proteome, and metabolome, obtaining more detailed information is possible, without the contamination generated by other cell types. Laser microdissection techniques used to isolate plant cells are powerful tools when coupled with high-throughput analyses. For example, peltate glandular trichomes of *Leonurus japonicus* were isolated with laser microdissection and, after analysis with ultra-performance liquid chromatography-tandem mass spectrometry (UPLC-MS/MS), two labdane diterpenoids (leoheterin and galeopsin) with anti-inflammatory activity were identified [67].

We will not review here all the literature on the transcriptome-proteome-metabolome of isolated trichomes, as excellent reviews have already been published [68,69]. We only provide a few recent examples dealing with the application of -omics on isolated trichomes. -Omics have been applied on trichomes isolated from several plant species. For example, a study on tomato highlighted the connection between primary metabolism and the production of secondary metabolites [70]. The combination of the different high-throughput techniques enabled the drawing of a model explaining the interconnection between carbon metabolism and the supply of precursors needed for secondary metabolite biosynthesis. Sucrose imported from the leaves is the chief carbon source and the light-dependent photosynthetic reactions provide energy and reducing equivalents. Interestingly, this energy and reducing power is aimed at the synthesis of lipids and terpenoids. Performing similar studies on the trichomes of local tomato varieties with different bioactive abundances would help understand, from a molecular point of view, the reasons for such differences.

## 5. Future Perspectives

**Figure 1:**
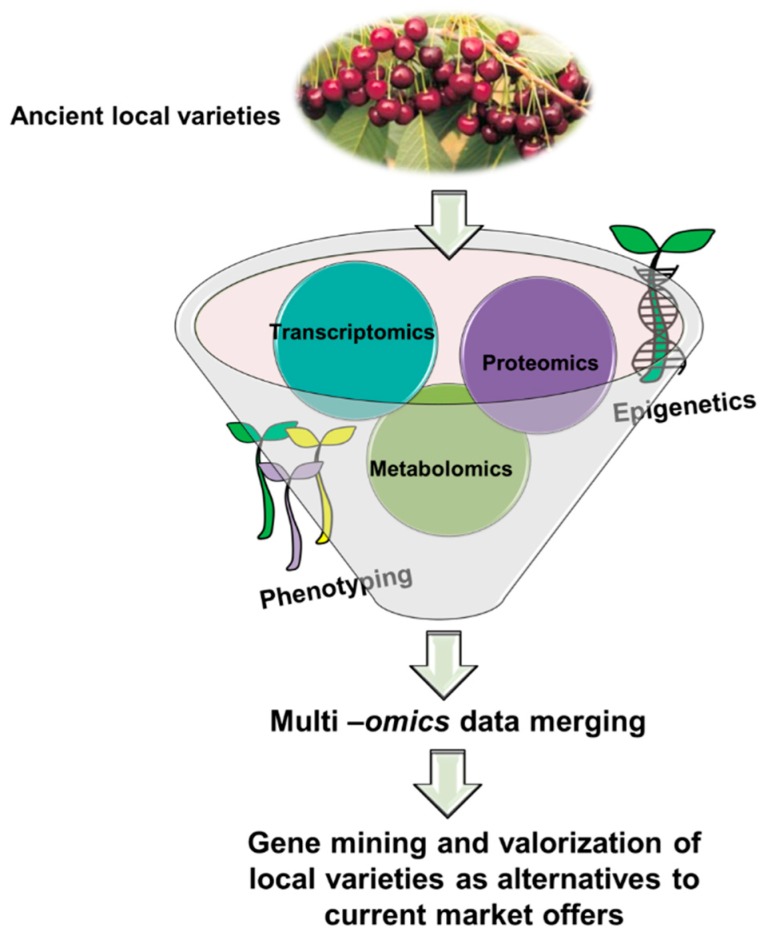
Multi-pronged approach on ancient local varieties. The sweet cherry variety Morellona is shown; image taken from the repository (http://germoplasma.regione.toscana.it/index.php?option=com_content&view=article&id=4&Itemid=109). Such an approach can combine -omics with epigenetics and phenotyping to find genes of interest that could then be used for biotechnological applications. The ultimate goal is to promote local varieties as alternatives to the current market of fruit and vegetables.

Innovation in the agricultural field is one of the most important topics in the scientific community. Finding new and possibly sustainable agronomic techniques to improve productivity and resistance to climate factors is fundamental to meet the ever-growing commercial demand. Furthermore, cultivation strategies are being developed in the field of green technology. This research field is worth increased scientific attention, because it guarantees products with improved commercial characteristics, such as productivity, taste, and content of health-promoting compounds, and a minimal impact on the environment. Future efforts should be devoted to the analysis, via systems biology approaches merging -omics, of underutilized varieties, such as ancient local varieties, to compare their content of bioactive molecules with respect to that found in commercial ones and to understand the molecular basis of such differences. -Omics can be merged with epigenetics and phenotyping (Figure 1) to explain the molecular details underlying the expression of specific characteristics in ancient local varieties. Such studies favor the diversification of the current market of fruit and vegetables and promote programs aimed at the preservation of regional agrobiodiversity.

## Figures and Tables

**Table 1:** Summary of some studies addressing the quantification of bioactives in ancient local varieties of herbaceous and woody species.

Type	Species	Key Features	Reference
Herbaceous	*Triticum* spp.	Higher lutein content in einkorn	[23]
Herbaceous	*Triticum spelta*	High amounts of bioaccessible and bioavailable free phenolic acids	[22]
Herbaceous	*Brassica oleracea*	High amounts of glucosinolate, carotenoid and polyphenols in broccoli; high levels of vitamin C in cauliflower	[25]
Herbaceous	*Brassica* spp.	Colored cauliflower from Sicily display higher content of aliphatic glucosinolates	[26]
Woody	*Olea europaea*	High PUFA content in the old accessions Salella	[27]
Woody	*Vitis vinifera*	In terms of bioactive content, ranking strictly depending on the class of phenolics considered	[28]
Woody	*Malus domestica*	High content of flavanols and phenolic acids in the flesh, while no major differences in the peels	[29]
